# Granzyme B; the chalk-mark of a cytotoxic lymphocyte

**DOI:** 10.1186/1479-5876-2-36

**Published:** 2004-10-25

**Authors:** Nigel J Waterhouse, Karin A Sedelies, Chris JP Clarke

**Affiliations:** 1 Cancer Immunology Program, Peter MacCallum Cancer Centre, Locked Bag 1, A'Beckett Street, Melbourne, 8006, Australia

## Abstract

During cytotoxic lymphocyte (CL) mediated killing of target cells, granzyme B is released from the CL into the immune synapse. Recent studies have found that ELISPOT-detection of granzyme B correlated well with conventional assays for CL mediated killing. In this way, the released granzyme B can be used to mark the spot where a target cell was murdered. We discuss the benefits and potential limitations of using this assay to measure CL mediated killing of target cells.

## Introduction

Cytotoxic Lymphocytes (CLs) eliminate virally infected cells or tumour cells either by activating death receptors or by delivering cytotoxic granule proteins (granule exocytosis) to the target cell [1,2]. The ability of a virus or a tumour cell to evade detection or survive an attack by CLs is likely to result in a more aggressive disease. The ability to measure specific killing of target cells by CLs is therefore of great interest to clinicians and researchers alike. Any assay for CL-induced death involves mixed cultures of target and effector cells and must include some means of distinguishing between the two. The current approach is to measure the release of a label, such as ^51^Cr or, more recently calcein-AM [3], that has been preloaded into the target cells. Radioactivity limits the utility of ^51^Cr and, although this type of assay is presumed to measure rupture of the plasma membrane (cell lysis), it is not formally known what is being measured.

## Discussion

Various alternative assays have been developed to assay CL-induced killing of target cells [4-10], however ^51^Cr remains the gold standard. Recently, Shafer-Weaver *et al *and others have utilized an interesting strategy aimed at measuring the functions of effector cells rather than death of the target cell [9,11]. During granule-mediated killing, granule enzymes (granzymes) are transferred to the target cell [2,12]. In the target cell granzyme B, can initiate target cell death by apoptosis [13,14]. Shafer-Weaver *et al*., [11] demonstrated that detection of granzyme B by ELISPOT correlated well with ^51^Cr release during antigen specific target cell death induced by cytotoxic T-lymphocytes and now report utility of this assay for measuring MHC non-restricted killing by natural killer cells [15]. Following incubation of CL with their targets, *Shafer-Weaver et al*., measured granzyme B by ELISPOT and found that the number of SPOTS correlated well with results obtained by the ^51^Cr release assay. Unlike the ^51^Cr release assay, this ELISPOT assay measures a specific and well-characterized event that occurs following target recognition. Assessing granzyme B by ELISPOT appears superior to other markers, such as IFNγ, because it assays a molecule that directly participates in CL mediated killing. Furthermore, the assay is non-radioactive and under the experimental parameters reported, it appears possible to detect cytolytic activity using fewer cells than are required for ^51^Cr release.

This assay appears to provide an effective alternative method for assessing CL-mediated cell death, however, users should be aware of possible limitations. The assay measures granzyme B release, not cell death. Frequently, the two will be closely correlated, but under certain circumstances using granzyme B release as a marker could lead either to an under or over estimate of target cell death. For example, perforin-deficient CLs are unable to kill targets [16,17], yet they may release granzyme B in the same way as wild type cells -leading to a false positive result. Alternatively, cells lacking, or expressing small amounts of granzyme B may retain the ability to kill targets by means of other granule components or through death receptor mediated pathways leading to an underestimate of cytotoxic activity [18]. In addition, a CL may degranulate normally, but certain targets may be inherently resistant to their effects [19]. Thus, to be certain that degranulation is inducing target cell death, chromium release assays should be performed alongside the granzyme B ELISPOT.

The limits of detection of this assay are not clear. It is not known whether the granzyme B released at a single death-inducing synapse are sufficient to produce a spot or whether a CL must degranulate several times, possibly killing multiple targets, to facilitate detection. Even if one spot reflects degranulation by one CL and is directly equivalent to one target cell death, it remains possible that CLs expressing granzyme B below the level of detection by ELISPOT may express sufficient granzyme B to kill their targets. These are difficult issues to address, but the correlation between ^51^Cr and granzyme B ELISPOT shown under the conditions used by *Shafer-Weaver et al *[15] suggests that the levels of detection of the assay are likely to be broadly equivalent to those required for cell death. It is however too difficult to directly compare these two assays. For example, 316 spots were detected in an assay using 50,000 target cells and 10,000 effectors (Table 1). This is equivalent to 0.6 +/- 0.1 % (as the number of spots must be related to the number of targets for comparison with ^51^Cr). Increasing the effectors generated too many spots to count. Therefore an experiment optimised for ^51^Cr assay, (0–70% release as reported in Table 1), will only have a dynamic range of between 0 and 0.6% using the ELISPOT assay. In contrast, an assay optimized for ELISPOT is likely to be off scale in a ^51^Cr release assay. These data suggest that a small amount of killing (e.g in a sample with low level killing) may easily generate a positive result by ELISPOT. It is therefore likely that stringent titration of both effectors and targets over a narrow range will be essential.

## Conclusion

The granzyme B-ELISPOT introduces a new assay for measuring CL mediated toxicity that will have a widespread utility in experimental systems where granzyme B is present in the effector cell and the target is susceptible to CL mediated killing. However, no assay used in isolation can be the answer to everyone's prayers and the granzyme B ELISPOT, like all others, has limitations. There is no doubt that this assay measures triggering of degranulation, but it does not directly address the question of cell death. Therefore it is likely that the greatest utility of this assay will be found by using it in combination with other existing measures of cytotoxic activity. It may also be extremely valuable as a quick reference to determine whether killing can occur in an assay with defined targets and effectors.

## Abbreviations

CL, cytotoxic lymphocytes; ELISPOT, enzyme linked immunospot; Cr, Chromium;

## Competing Interests

The authors declare that they have no competing interests.

## Author's contributions

All authors contributed to the ideas, discussion and preparation of this manuscript.
